# Agreement and trending ability between non-invasive and invasive hemodynamic monitoring during cesarean section under spinal anesthesia: a prospective observational study

**DOI:** 10.3389/fmed.2026.1870822

**Published:** 2026-07-29

**Authors:** Yongqiang Shi, Yueji Ma, Jiawei Cun, Xinxin Yu, Yi Chen

**Affiliations:** 1 Department of Anesthesiology and Perioperative Medicine, General Hospital of Ningxia Medical University, Yinchuan, China; 2 School of First Clinical Medical, Ningxia Medical University, Yinchuan, China

**Keywords:** Cesarean Section, FloTrac/Vigileo, hemodynamic, NICAP/NICCO, spinal anesthesia

## Abstract

**Background:**

The uteroplacental circulation lacks autoregulatory capacity and therefore relies predominantly on the maternal cardiac output (CO). Monitoring hemodynamic parameters such as CO can provide guidance for anesthesia decisions during cesarean section. We evaluated the agreement and trending ability of maternal CO, stroke volume (SV), and stroke volume variation (SVV) between two clinically used hemodynamic monitoring technologies, the non-invasive NICAP/NICCO system and the invasive FloTrac/Vigileo system, in patients undergoing elective cesarean section under spinal anesthesia.

**Methods:**

Forty-five patients were included in the final analysis. Prior to anesthesia, the patients’ hemodynamic parameters, including CO, SV, and SVV, were simultaneously monitored using both the NICAP/NICCO and Flotrac/Vigileo. The baseline values and continuous dynamic data of CO, SV, and SVV were collected over a 15-min period following spinal anesthesia and recorded at 20-s intervals. Repeated-measures Bland–Altman analysis, percent error (PE) and ICC (2, 1) were employed to assess the agreement between the two monitoring methods. Trending ability was evaluated using four-quadrant plot analysis.

**Results:**

The repeated-measures Bland–Altman analysis indicated that the biases of CO, SV, and SVV were −0.89 L/min (95% limits of agreement: −4.08 to 2.29 L/min; PE, 51.24%), −10.53 mL (95% limits of agreement: −46.26 to 25.20 mL; PE, 46.84%), and 2.44% (95% limits of agreement: −6.82 to 11.69%; PE, 77.95%), respectively. The results of the ICC indicated that the values of CO, SV, and SVV were 0.2578 (95% CI: 0.1067 to 0.3772), 0.4682 (95% CI: 0.2397 to 0.6111), and 0.1813 (95% CI: 0.0686 to 0.2779), respectively. Four-quadrant plot analysis demonstrated the concordance rates for CO, SV, and SVV were 44.59, 32.39, and 21.48%, respectively.

**Conclusion:**

During cesarean section under spinal anesthesia, the non-invasive hemodynamic monitoring employing NICAP/NICCO technology exhibits poor agreement and trending ability with the invasive hemodynamic monitoring utilizing Flotrac/Vigileo technology.

**Clinical trial registration:**

ClinicalTrials.gov, identifier NCT06473818

## Introduction

Owing to the extensive sympathetic nerve block subsequent to spinal anesthesia, the maternal arteries undergo dilation, and the peripheral vascular resistance declines (1). As a result, hypotension emerges as a prevalent complication during cesarean section, with an incidence rate reaching up to 62.1% (2). Hypotension can induce maternal nausea and vomiting and may even impact the uteroplacental circulation and neonatal outcomes (3). The uteroplacental circulation lacks autoregulatory capacity and therefore relies predominantly on the maternal CO (4). Consequently, monitoring hemodynamic parameters, such as CO, can offer guidance for anesthesia decisions, including the rational utilization of vasopressors and the optimization of fluid management during cesarean section (5, 6).

In recent years, invasive hemodynamic monitoring based on arterial pulse wave contour analysis, such as the Flotrac/Vigileo technology (e.g., EV1000 system; Edwards), has been extensively utilized in perioperative hemodynamic monitoring to acquire real - time and continuous parameters such as CO, stroke volume (SV), and stroke volume variation (SVV) (7–9). Nevertheless, due to the risks of complications such as trauma and infection, along with the high cost, its routine application of this technology in patients undergoing cesarean section is restricted. Currently, non-invasive hemodynamic monitoring techniques, including NICAP/NICCO technology (pulse wave velocity + waveform contour analysis method), possess advantages such as convenience, rapidity, real-time monitoring, and non-invasiveness, and are gradually being utilized in obstetric anesthesia (10, 11). However, the agreement and trending ability of these techniques with traditional monitoring methods have not been thoroughly evaluated.

We evaluate the agreement and trending ability of maternal CO, stroke volume (SV), and stroke volume variation (SVV) between non - invasive and invasive hemodynamic monitoring, which are based on NICAP/NICCO and Flotrac/Vigileo technology respectively, during elective cesarean section under spinal anesthesia.

## Methods

This study is a single - center, prospective observational study that has been approved by the Ethics Committee of Ningxia Medical University General Hospital (No.: KYLL-2024-0384) and prospectively registered on ClinicalTrials.gov (No. NCT06473818). The study was carried out from November 2025 to January 2026. All patients provided written informed consent prior to the monitoring of hemodynamic parameters. The inclusion criteria were as follows: patients aged between 18 and 45 years, with an ASA grade of II, and single full-term pregnancy undergoing elective cesarean section under spinal anesthesia. The exclusion criteria were as follows: BMI equal to or greater than 40 kg/m^2^, preeclampsia or chronic hypertension or baseline systolic blood pressure equal to or greater than 140 mmHg, hemoglobin less than 7 g/dL, fetal distress, and patients with known fetal developmental abnormalities.

All patients received routine care and were monitored for electrocardiogram (ECG), non-invasive blood pressure (NIBP), and transcutaneous pulse oxygen saturation (SpO_2_) upon entering the room. A peripheral venous access (18G indwelling needle) was established in the upper limb for fluid infusion and the administration of vasopressors. A bolus of 75 μg of phenylephrine was administered intravenously when the systolic blood pressure (SBP) measured by the FloTrac/Vigileo system fell below 80% of the baseline value. This bolus could be repeated as needed until SBP recovered to≥ 80% of baseline. All patients received a crystalloid coload of 10 mL/kg of lactated Ringer’s solution.

All patients underwent both non-invasive and invasive hemodynamic monitoring simultaneously. Non-invasive hemodynamic monitoring was performed using the NICAP/NICCO system (T20A, Chongqing, China). This system employs NICAP technology to derive pulse transit time (PTT) and pulse wave velocity (PWV) from dual-channel pulse wave signals (posterior auricular and dorsal pedal arteries), combined with morphological analysis to estimate blood pressure. Simultaneously, NICCO technology, based on pulse wave area and waveform contour analysis, calculates stroke volume (SV), with cardiac output (CO) derived as SV × heart rate. A one-time baseline calibration was performed using each patient’s demographic data (age, height, and weight), and no recalibration was required during the 15-min recording period. Signal quality was continuously monitored, and data segments with visible artifacts were excluded. Invasive hemodynamic monitoring was performed using the FloTrac/Vigileo system (Version 4.00, Edwards Lifesciences, Irvine, CA, United States). Following radial artery cannulation, the pressure transducer was zeroed and calibrated to ensure a clear and undiminished arterial waveform. CO, SV, and SVV data were concurrently collected, and the influence of position changes and external interference on signal collection was precluded. The time axes of the both monitoring were precisely calibrated to ensure full synchronization of the data sequence. All parameters were recorded at intervals of 20 s, and the average value of the continuous 3-min measurement during the patient’s resting state was taken as the baseline value. Continuous recording commenced immediately after spinal anesthesia and lasted for 15 min.

The patient was positioned in the lateral decubitus position. A 25-gauge puncture needle was employed to conduct a subarachnoid puncture at the L3–4 intervertebral space. Upon confirmation of the outflow of cerebrospinal fluid, 12.5 mg of bupivacaine (0.5% w/v) was injected. Immediately following spinal anesthesia, the operating table was inclined 15° to the left. The level of sensory block was assessed by testing for cold and sharp pain sensations. The surgical procedure was initiated when the sensory block reached T6 or higher.

The primary endpoint was the agreement and trending ability of CO, SV and SVV measured by NICAP/NICCO and Flotrac/Vigileo. The secondary endpoints included: the incidence of hypotension (systolic blood pressure less than 80% of the baseline value), severe hypotension (systolic blood pressure less than 60% of the baseline value), nausea and vomiting, bradycardia (heart rate less than 60 beats per minute), and hypertension (systolic blood pressure greater than 120% of the baseline value or equal to or greater than 160 mmHg); the total vasopressor consumption in each patient was documented; neonatal umbilical artery blood gas values (pH, PaCO₂, PaO₂, base excess) and the incidence of pH less than 7.2; Apgar scores (scores at 1 min and 5 min).

## Sample size determination and statistical analysis

According to the protocol of the Association for the Advancement of Medical Instrumentation (AAMI), a minimum of 35 patients are necessary to validate new monitoring tools for specific populations (12).

The Kolmogorov–Smirnov test was employed to examine the normality of continuous variables. Continuous variables that adhered to a normal distribution were presented as the mean ± standard deviation; those that did not adhere to a normal distribution were presented as the median [interquartile range]; categorical variables were presented as numbers (%). Agreement between the two monitoring methods was assessed using repeated-measures Bland–Altman analysis (mean bias, 95% limits of agreement) and ICC (2, 1) analysis (two-way random effects model with the same raters for all subjects). Trending ability was evaluated using four-quadrant plot analysis. Sequential changes (*Δ*) in CO, SV, and SVV between each adjacent 20-s recording point were calculated for both methods. Four-quadrant plots were constructed by plotting the changes measured by NICAP/NICCO on the y-axis against those measured by FloTrac/Vigileo on the x-axis. The exclusion zone was set at 10% of the mean value for each parameter. A concordance rate of ≥92% was predefined as the clinically acceptable threshold for trending ability. The percentage error (PE) was computed using the formula PE = 1.96SD (diff)/(Mean A + Mean B)/2 × 100%, and a PE ≤ 30% was regarded as clinically acceptable (13). The data were analyzed using SPSS 26.0 and MedCalc 20.0 statistical software. A two-sided *p* < 0.05 was deemed statistically significant.

## Results

**Figure 1:**
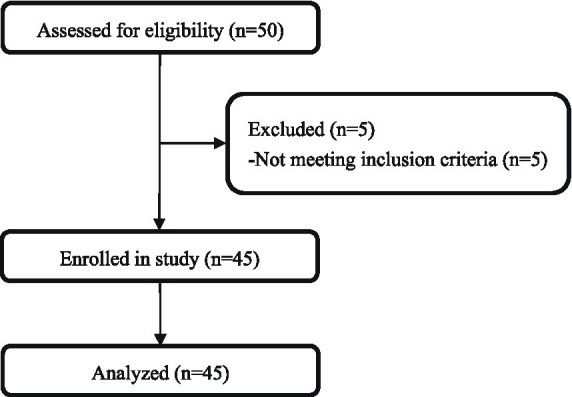
Flow chart.

A total of 45 patients were incorporated into the final analysis (Figure 1), and all the monitoring data were comprehensive and valid. The baseline characteristics of the patients are presented in Table 1.

**Table 1:** The baseline characteristics.

Variable	Population (*n* = 45)
Age (years)	31.93 ± 6.16
Height (cm)	162.07 ± 5.06
Weight (kg)	74.80 ± 9.65
BMI (kg/m^2^)	28.42 ± 3.10
Gestational weeks (weeks)	39 [38, 40]
Level of sensory block	T6 [T6, T6]
The time from anesthesia to fetal delivery (min)	14.76 ± 2.59
The time from incision to fetal delivery (min)	3.51 ± 1.49
Baseline systolic blood pressure (mmHg)	126.31 ± 9.98
Baseline heart rate (bpm)	91.16 ± 11.96

Data are presented as mean ± SD (standard deviation) and median [interquartile range].

A comparison of the baseline values of NICAP/NICCO and Flotrac/Vigileo in the resting state prior to anesthesia indicated that there was no statistical significance in CO and SV. For NICAP/NICCO, the SVV was higher than that of Flotrac/Vigileo (*p* = 0.002), as presented in Table 2.

**Table 2:** Comparison of the baseline values between NICAP/NICCO and Flotrac/Vigileo for CO, SV, and SVV.

*n* = 45	Flotrac/Vigileo (mean ± SD)	NICAP/NICCO (mean ± SD)	Mean difference	95%CI of difference	*p*-value
CO (L/min)	6.44 ± 1.16	6.40 ± 1.01	0.03	−0.40 to 0.46	0.878
SV (ml)	70.29 ± 15.50	70.27 ± 7.92	0.22	−4.47 to 4.51	0.992
SVV (%)	11.84 ± 2.52	13.71 ± 3.20	−1.87	−3.03 to −0.70	0.002

CO, cardiac output; SV, stroke volume; SVV, stroke volume variation; SD, standard deviation; CI, confidence interval. Mean difference = FloTrac/Vigileo − NICAP/NICCO.

**Figure 2:**
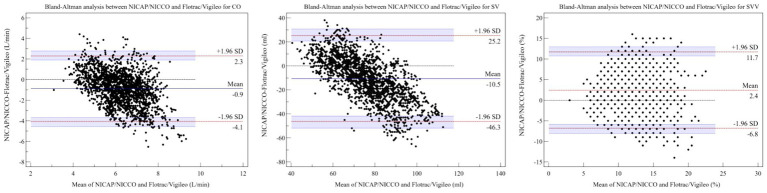
Repeated-measures Bland–Altman analysis between NICAP/NICCO and FloTrac/Vigileo for cardiac output (CO), stroke volume (SV), and stroke volume variation (SVV). The analysis included 2,070 paired observations from 45 patients.

**Figure 3:**
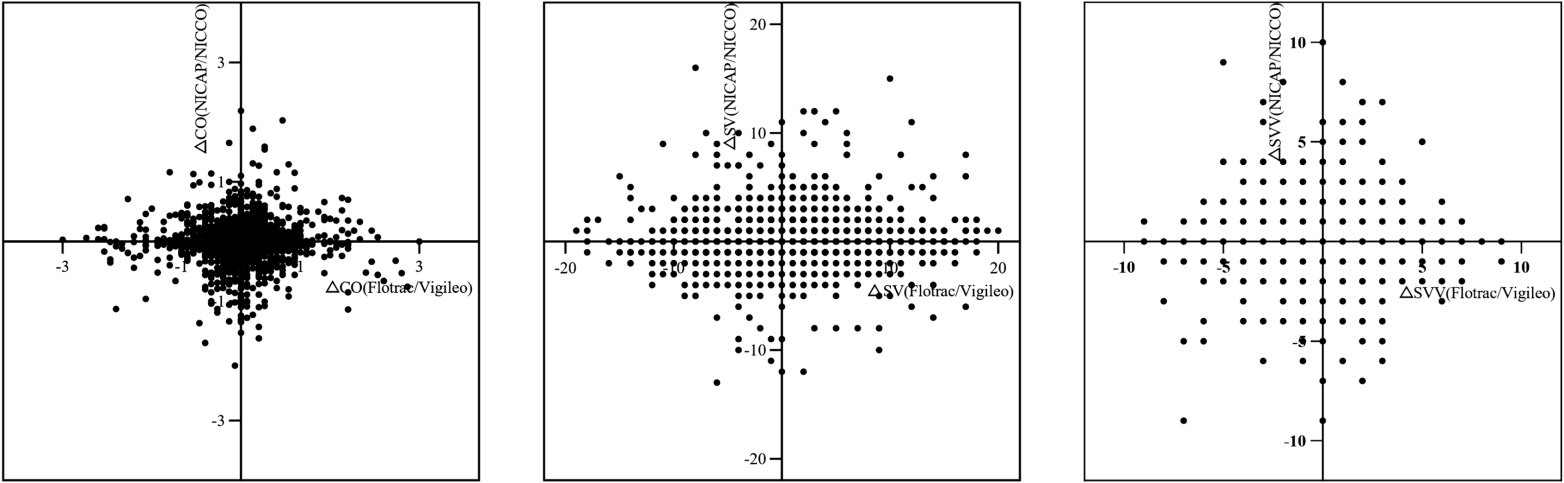
Four-quadrant plot analysis between NICAP/NICCO and FloTrac/Vigileo for cardiac output (CO), stroke volume (SV), and stroke volume variation (SVV). The exclusion zone was set at 10% of the mean value for each parameter. A concordance rate of ≥ 92% was predefined as the clinically acceptable threshold for trending ability.

A total of 2070 data points including CO, SV, SVV, were collected for analysis. The repeated-measures Bland–Altman analysis indicated that the biases of CO, SV, and SVV were −0.89 L/min (95% limits of agreement: −4.08 to 2.29 L/min; PE, 51.24%), −10.53 mL (95% limits of agreement: −46.26 to 25.20 mL; PE, 46.84%), and 2.44% (95% limits of agreement: −6.82 to 11.69%; PE, 77.95%), respectively, as presented in Figure 2. The results of the ICC indicated that the values of CO, SV, and SVV were 0.2578 (95% CI: 0.1067 to 0.3772), 0.4682 (95% CI: 0.2397 to 0.6111), and 0.1813 (95% CI: 0.0686 to 0.2779), respectively. Four-quadrant plot analysis demonstrated the concordance rates for CO, SV, and SVV were 44.59, 32.39, and 21.48%, respectively, all far below the clinically acceptable threshold of 92% for trending ability, as presented in Figure 3.

The maternal and neonatal outcomes are shown in Table 3.

**Table 3:** Maternal and neonatal outcomes.

Variable	Population (*n* = 45)
Maternal outcomes	
Hypotension, *n* (%)	28 (62.22)
Severe hypotension, *n* (%)	5 (11.11)
Hypertension, *n* (%)	8 (17.78)
Bradycardia, *n* (%)	8 (17.78)
Nausea and vomiting, *n* (%) Total vasopressor consumption (μg)	3 (6.67) 75 [0, 225]
Neonatal outcomes	
pH	7.32 ± 0.05
PCO_2_ (mmHg)	45.50 ± 6.12
PO_2_ (mmHg)	21.82 ± 5.08
BE (mmol/L)	−3.42 ± 1.36
Apgar at 1 min	9 [9, 9]
Apgar at 5 min	10 [10, 10]

Data are presented as number (%), mean ± SD (standard deviation) and median [interquartile range].

## Discussion

The findings of this study indicate that the agreement and trending ability between the NICAP/NICCO and FloTrac/Vigileo systems for hemodynamic monitoring are poor. NICAP/NICCO demonstrated a fixed systematic bias, characterized by underestimation of CO and SV and overestimation of SVV, with percentage errors exceeding 30%, wide 95% limits of agreement, and concordance rates all below the clinically acceptable threshold of 92%.

FloTrac/Vigileo is a commonly used hemodynamic monitoring system with applications in obstetric anesthesia. It acquires core hemodynamic parameters via peripheral arterial waveform analysis in a minimally invasive manner. Previous studies have suggested that its algorithm may accommodate the hemodynamic characteristics of pregnancy—including increased cardiac output, decreased systemic vascular resistance, and expanded blood volume—and may provide clinically valuable assessments of maternal hemodynamic status (14–16). In our study, the maternal baseline CO was 6.44 ± 1.16 L/min, and the SV was 70.29 ± 15.50 mL, which was consistent with the CO of 5.50 ± 0.99 L/min and SV of 69.41 ± 15.78 mL reported by Gao et al. (17) using the Flotrac/Vigileo. Additionally, when continuous hemodynamic monitoring was conducted based on the LiDCOplus, the baseline CO was 7.0 ± 0.4 L/min, and SV was 79 ± 5 mL (18), which was similar to the results of Flotrac/Vigileo monitoring in our study, indicating that Flotrac/Vigileo can serve as a clinical reference for NICAP/NICCO. Notably, FloTrac/Vigileo itself is an indirect estimation technique based on arterial pulse contour analysis, and cannot be regarded as the gold standard for cardiac output measurement. Pulmonary artery thermodilution remains the traditional reference method for cardiac output measurement (19). In addition, transpulmonary thermodilution or transthoracic echocardiography could provide more robust validation for non-invasive monitoring technologies (20, 21). Our study only evaluates the agreement and trending ability between two clinically available hemodynamic monitoring technologies, rather than verifying the absolute accuracy of NICAP/NICCO against a reference standard. The findings of this study are intended to provide reference for the clinical interchangeability of the two methods in obstetric anesthesia practice.

The results of the repeated-measures Bland–Altman analysis indicate that NICAP/NICCO exhibits a fixed systematic bias, which is characterized by a significant underestimation of CO and SV and an overestimation of SVV. In our study, the PE values all substantially exceeded the 30% threshold, and the concordance rates were consistently below 92%, indicating poor agreement and trending ability between the two devices. Although the 30% threshold was derived from general surgical populations with a mean cardiac output of approximately 5 L/min, the clinically unacceptable disagreement observed in our study is primarily attributable to inherent measurement differences between the two devices. Nevertheless, there is no significant disparity between NICAP/NICCO and Flotrac/Vigileo in the monitoring of CO, SV, and SVV during the pre - anesthesia baseline state, suggesting that non-invasive monitoring holds good reference value when the circulation is stable and there are no significant alterations in vascular tone. However, the deviation significantly widens during the hemodynamic fluctuation period following spinal anesthesia, indicating that non-invasive monitoring has low accuracy during periods of rapid circulatory changes. Given that the hemodynamic fluctuation period following spinal anesthesia is a crucial phase for anesthesia management, the significant bias in hemodynamic monitoring by NICAP/NICCO will further impact the accuracy of its assessment. Additionally, Bonnin et al. (11) concurrently monitored CO in 100 pregnant women in the late stage of pregnancy using non-invasive (finger volume plethysmography) and transthoracic echocardiography, and also demonstrated that non-invasive monitoring has poor reliability. Therefore, NICAP/NICCO and FloTrac/Vigileo cannot be used interchangeably for clinical decision-making during cesarean section under spinal anesthesia.

This study found that the deviation between NICAP/NICCO and Flotrac/Vigileo was closely associated with the monitoring principles, patient physiology, and intraoperative interference factors. First, Flotrac/Vigileo obtains the pulse wave contour through direct radial artery pressure measurement, which offers stable signals and strong anti-interference capabilities. In contrast, NICAP/NICCO is based on thoracic electrical bioimpedance and non-invasive pulse wave deduction calculation, which is vulnerable to factors such as respiratory movement, body movement, chest thickness, subcutaneous fat, skin impedance, and electrode position (22). Moreover, the signal stability significantly declines in the intraoperative environment. Second, during the late stage of pregnancy, the maternal circulation is in a state of higher volume, higher CO, and lower peripheral vascular resistance. After spinal anesthesia, sympathetic block further causes rapid peripheral vasodilation. The built-in vascular resistance correction model in non-invasive monitoring cannot match the patient’s rapidly changing circulatory state in real-time, thus resulting in a systematic underestimation of CO and SV (23). Third, SVV is a dynamic volume responsiveness parameter, which has extremely high requirements for respiratory waveforms, pulse signal quality, and body position stability. In cesarean section, changes in body position, surgical traction, abdominal pressure changes, and maternal stress breathing can all cause distortion of non - invasive waveforms, leading to an overestimation of SVV. The aforementioned factors collectively lead to stable and repeatable deviations between NICAP/NICCO and Flotrac/Vigileo.

Intraoperative blood pressure management was aimed at maintaining the maternal blood pressure above 80% of the baseline value. The overall incidence of hypotension was in line with the findings of previous clinical research. Nevertheless, the incidences of severe hypotension (7.8%), hypertension (2.4%), and bradycardia in this study were higher than those reported in the previous literature (24, 25). The reasons are as follows: This study used continuous real-time hemodynamic monitoring with a 20-s sampling interval. Compared with the conventional 1-min intermittent cuff blood pressure measurement used in previous studies, this method has a higher time resolution and is more sensitive in capturing transient hemodynamic fluctuations. As a result, it can more accurately identify concealed hypotension, severe hypotension, and transient blood pressure elevation events after spinal anesthesia, leading to a relatively higher detection rate of abnormal circulatory events in this group. Moreover, the neonatal outcomes, such as umbilical artery blood gas analysis and Apgar score, were all within the normal range (26, 27).

This study presents the following limitations: First, the sample size of this study is relatively small, and only full-term patients with ASA grade II were included. As a result, the generalizability of the results to high-risk patients, those with multiple pregnancies, obese patients, and patients with gestational hypertension is limited. Second, this study employed rescue treatment for maternal hypotension, which is inconsistent with the current clinical practice of preventive application of vasopressors (28). In the future, ensuring better hemodynamic stability under the application of prophylactic vasopressors infusions may have clinical application value for further evaluation of NICAP/NICCO during cesarean section. Third, we used FloTrac/Vigileo as the clinical reference, rather than a recognized gold standard method such as pulmonary artery thermodilution. Therefore, our findings reflect only the agreement between these two monitoring technologies and cannot be used to determine the absolute accuracy of NICAP/NICCO. Future studies employing transpulmonary thermodilution or echocardiography as reference methods are warranted to further validate the performance of non-invasive hemodynamic monitoring in the obstetric population. In addition, we did not use mixed-effects models for analysis; future studies employing such methods may further clarify factors affecting inter-device agreement.

In conclusion, during cesarean section under spinal anesthesia, the non-invasive hemodynamic monitoring employing NICAP/NICCO technology exhibits poor agreement and trending ability with the invasive hemodynamic monitoring utilizing Flotrac/Vigileo technology.

## Data Availability

The original contributions presented in the study are included in the article/supplementary material, further inquiries can be directed to the corresponding author.
